# Analysis of Phospholipids in Digestion Using Hybrid IDA and SWATH Acquisition: An Example for Krill Oil

**DOI:** 10.3390/foods12102020

**Published:** 2023-05-16

**Authors:** Jiachen Shi, Yanan Wang, Yuanfa Liu, Yongjiang Xu

**Affiliations:** State Key Laboratory of Food Science and Technology, School of Food Science and Technology, National Engineering Research Center for Functional Food, National Engineering Laboratory for Cereal Fermentation Technology, Collaborative Innovation Center of Food Safety and Quality Control in Jiangsu Province, Jiangnan University, 1800 Lihu Road, Wuxi 214122, China

**Keywords:** phospholipid-rich foods, digestion, LC-MS, model-assisted, predicted retention time

## Abstract

The composition and digestion of phospholipid-rich foods have important effects on the health of the body. Herein, a model-assisted liquid chromatography coupling mass spectrometry (LC-MS) method was established to analyze the phosphatidylcholine (PC) and lyso-phosphatidylcholine (LPC) species in krill oil before and after digestion. According to the confirmed PC and LPC species in the IDA (information dependent acquisition) results, three categories of mathematical models were set up, involving the retention time (RT), carbon number and unsaturation degree of the fatty acyl chain. All of the regression coefficient values (*R*^2^) were greater than 0.90, showing satisfactory fitting results. On this basis, using the computationally created precursor ion mass of PC and LPC species, 12 extra PC species and 4 LPC species were found in the SWATH (sequential windowed acquisition of all theoretical fragment ions) results. The PC and LPC compositions in the final digestive products had obvious differences among the different krill oils with different phospholipid content. Furthermore, more than half of the LPC species in the final digestive products were newly generated, indicating that LPC was one of basic constituents in the digestive products of krill oil. In conclusion, model-assisted hybrid IDA and SWATH acquisition has excellent detection performance, contributing to deep studies of the formations and functions of phospholipids.

## 1. Introduction

Phospholipids, as an essential class of lipids, can be subdivided into many subclasses, such as phosphatidylcholine (PC), phosphatidylethanolamine (PE), phosphatidylserine (PS), phosphatidylglycerol (PG), phosphatidylinositol (PI) and corresponding lyso-phospholipids. All of these phospholipids have specific molecular structures and biological functions [1,2]. As one of the significant indicators of food nutrition, monitoring phospholipid composition and digestion in foods is of great attention. Using an in vitro digestion model, the digestion of different edible oils was investigated by referring to the comparison of different triacylglycerol compositions, different carbon numbers of fatty acyl chains and different unsaturation degrees of fatty acyl chains [3,4]. However, few reports concerning phospholipid-rich oils have been published, especially those focusing on alterations to digestive behaviors caused by different phospholipid content.

As for the analysis of phospholipid compositions, relevant methods have been continually developed, including high-performance liquid chromatography (HPLC), ^31^P nuclear magnetic resonance (^31^P-NMR), liquid chromatography coupling mass spectrometry (LC-MS), etc. [5,6,7]. Recently, the LC-MS method has become the most popular choice. In this method, the target molecules are generally acquired by triple quadrupole (QqQ) or Orbitrap instruments using the multiple reaction monitoring (MRM) mode, or by a time-of-flight mass spectrometer (TOF) with the information dependent acquisition (IDA) mode or sequential windowed acquisition of all theoretical fragment ion mass spectra (SWATH) mode [8,9,10]. Compared to the IDA and SWATH mode, the MRM mode has better sensitivity and selectivity, but a major restriction is the need for predefined ion pairs, limiting its application in the discovery of novel compounds [11].

Both IDA and SWATH modes obtain the signal at high resolution, while the former cannot acquire all fragment information because only the most *N*-abundant precursor ions would be dissociated. SWATH can acquire fragment information from all precursor ions, but the corresponding relations between precursor ions and fragment ions are weak, making it more difficult to identify the compounds [12]. For instance, Chen et al. used the IDA mode and acquired a lower false discovery rate than with the SWATH mode in the analysis of milk phospholipids, while Guo et al. applied the SWATH mode and optimized algorithms and recognized more metabolites than with the IDA mode in urine samples [13,14]. Krill oil is famous for its high phospholipid content (30–80% of total lipids), where PC species account for 60–96% of total phospholipids and relatively few PE species are present [15]. The detailed species have been analyzed using QqQ, Orbitrap and QToF-MS systems, but little attention is paid to the digestive behaviors and compositions of final digestive products [16,17,18].

In this study, we proposed a model-assisted hybrid IDA and SWATH acquisition strategy to analyze the phospholipids. Firstly, some mathematical models were established based on the confirmed species using the IDA mode, and the potential quasi-molecular ions created by the theoretical calculation were then extracted from SWATH results. The unknown species were ultimately identified according to the predicted retention time (RT) and characteristic product ions. Taking the krill oils for example, their composition in terms of PC and lyso-phosphatidylcholine (LPC) species was identified before and after digestion. Some novel species were discovered, illustrating the feasibility and sensitivity of this method and driving studies on phospholipid digestion at the molecular level.

## 2. Materials and Methods

### 2.1. Materials

Krill oil with 40%, 50% and 60% phospholipid content was obtained from Luhua Biomarine Co., Ltd. (Jinan, China), and some others were lab-made. The LC-MS grade reagents, such as acetonitrile (ACN), isopropanol (IPA) and ammonium acetate (CH_3_COONH_4_), were purchased from Thermo Fisher Scientific (Shanghai, China). The mucin from porcine stomach, pepsin from porcine gastric mucosa (250 U/mg), gastric lipase from *Rhizopus oryzae* (35 U/mg), porcine lipase (200 U/mg) and bovine bile were provided by Sigma Aldrich (Shanghai, China). Porcine trypsin (250 U/mg) and whey protein isolate (WPI) were supplied by Yuanye Bio-Technology Co., Ltd. (Shanghai, China) and Sinopharm Chemical reagent Co., Ltd. (Shanghai, China), respectively. Other analytical grade chemicals were also purchased from Sinopharm Chemical reagent Co., Ltd.

### 2.2. Sample Preparation

For phospholipid digestion, the 2 wt.% krill oil phase (containing different phospholipid content) and 98 wt.% aqueous phase (1.0 wt.% WPI, 5 mM phosphate buffer, pH 7.0) were made as emulsions. Specifically, the coarse emulsions were prepared using a high-speed blender (T18D S25, IKA, Staufen, Germany) for 3 min, and the emulsions were then homogenized using an ultrahigh-pressure homogenizer (AH2010, AST Nano Technology Co., Ltd., Suzhou, China) for another 3 min at 40 and 400 bar in the first and second stages.

For phospholipid species analysis, krill oils were dissolved in IPA solution to 0.1 mg/mL, and the upper layer was then injected into the LC-MS platform after centrifugation at 16,000× *g* for 10 min. When digestion was completed, the digestive products were mixed with chloroform and methanol (2:1, *v/v*) at a volume ratio of 1:5, followed by ultrasonication for 10 min. After centrifugation at 7200× *g* for 10 min, the organic fraction was dried under vacuum and phospholipids were contained in the residue. These extracts were resuspended and centrifuged for MS analysis.

### 2.3. In Vitro Digestion

The krill oil emulsions were passed through the in vitro static digestion model that simulated the mouth, stomach and intestine. The detailed procedures were performed according to the INFOGEST protocol with slight modifications [19].

Initial stage: The krill oil emulsions with different phospholipid contents were placed into an incubated shaker and heated to 37 °C for the further procedures.

Mouth phase: Simulated saliva fluid (SSF) containing 1.5 mM CaCl_2_ and 0.015 g/mL mucin was heated to 37 °C and mixed with the initial emulsion at a volume ratio of 1:1. After pH was adjusted to 6.8, the solution was incubated in the shaker for 2 min to mimic agitation in the mouth.

Stomach phase: The oral bolus was mixed with the simulated gastric fluid (SGF) containing 1.5 mM CaCl_2_, 2000 U/mL pepsin and 60 U/mL gastric lipase at the same volume. The pH was adjusted to 1.8 and stirred at 37 °C for 120 min to mimic stomach conditions.

Intestinal phase: The gastric chyme was also mixed with the same volume of simulated intestinal fluid (SIF) containing 0.6 mM CaCl_2_, 100 U/mL trypsin, 2000 U/mL pancreatic lipase and 10 mM bile salts. The solution was adjusted to pH 7.2 and incubated with agitation at 37 °C for 120 min. During this time, a pH-stat system was applied to monitor the pH and keep it at 7.2 via titration of 0.1 mM NaOH into the reaction system. The titration curves were recorded at the same time.

### 2.4. LC-MS Analysis for Phospholipids

Data acquisition for phospholipids was performed using RPLC coupled with the QToF-MS (5600^+^, AB SCIEX, Singapore) and operated in ESI positive mode.

The chromatographic processing was executed on an ExionLC AD system (AB SCIEX, Singapore), which was equipped with a Phenomenex Kinetex C18 column (100 mm × 2.1 mm, 2.6 μm). The separation was achieved using a gradient of mobile phases containing ultrapure water with 60% ACN and 10 mM CH_3_COONH_4_ (A Phase) as well as IPA with 10% ACN and 10 mM CH_3_COONH_4_ (B Phase). The mobile phase gradient was as follows: 0–1 min, 35% B; 1–14 min, 100% B; 14–16 min, 100% B; 16–16.5 min, 35% B; 16.5–18 min, 35% B. During the elution, the flow rate was 0.3 mL/min, column temperature was 40 °C, and injection volume was 3 μL.

The MS data acquisition was carried out in both IDA and SWATH modes. In the IDA mode, MS scan parameters were set as a mass range of 100–1000 Da and an accumulation time of 250 ms. Each scan was followed by 15 product ion scans with an accumulation time of 45 ms, and the mass range of the MS/MS scan was 80–900 Da. The ion source was set as gas 1 50 psi, gas 2 50 psi, curtain gas 40 psi, temperature 500 °C, declustering potential (DP) 90 V, spray voltage 5500 V and collision energy (CE) 40 ± 20 V. For SWATH mode, the mass range of precursor ions and the settings of the ion source were the same as with IDA, while the MS/MS scan was different. They were set to 15 acquisition windows (Table S1), and each window required 45 ms of accumulation time.

### 2.5. Determination of Particle Characterization

Particle size was measured using a multi-angle particle size analyzer (Nano Brook Omni, Brookhaven Instruments Corporation, Holtsville, NY, USA). All samples were diluted to a content of 0.03% (*wt./wt.*) with deionized water and particle size was illustrated as the surface-weighted mean diameter (*d*_3,2_, nm). The refractive index for water and particles was 1.330 and 1.492, respectively. Furthermore, particle size distribution was analyzed using a laser particle sizer (Microtrac, Shanghai, China), and the results are shown as a percentage of volume.

The *ζ*-potential was determined using the Zetasizer nano analyzer (Nano Brook Omni, Brookhaven Instruments Corporation, Holtsville, NY, USA). As with particle size analysis, samples were diluted to 0.03% and *ζ*-potential was determined at 25 °C. Both particle size and *ζ*-potential were observed in three replicate samples for each group, and all the samples were measured immediately after homogenization or digestion.

### 2.6. Data Processing and Statistical Analysis

Raw data (*.wiff) acquired with the IDA mode were submitted to MS-DIAL (version 4.80) for the identification of phospholipids. Following default parameters, the confirmed PC and LPC names and corresponding RT were collected. For data acquired using the SWATH mode, they were handled in OS-Q (version 1.7) to achieve peak detection. The confirmed phospholipid species were simultaneously based on three conditions: (a) precursor ion *m/z* extracted by the theoretical results; (b) MS/MS data having characteristic fragments (*m/z* at 184.07 for PC and LPC species); and (c) matching the models established on the RT, carbon number and unsaturation degree of the fatty acyl chain. Therein, the list of theoretical precursor ion mass was generated using R code (version 4.0.2), including the carbon number of 12 to 22 and unsaturation degree of 0 to 6 in a single fatty acyl chain. The deviations of precursor ion *m/z* and RT between predicted and practical results were accepted at less than 10 ppm and 15 s, respectively. The mathematical models were fitted and visualized with Origin (version 9.0). Other multivariate statistical analysis was applied using SIMCA-P (version 14.1), and variance analysis was executed using IBM SPSS Statistics (version 19.0).

## 3. Results and Discussion

### 3.1. Identification of PC and LPC Species Using IDA

**Figure 1:**
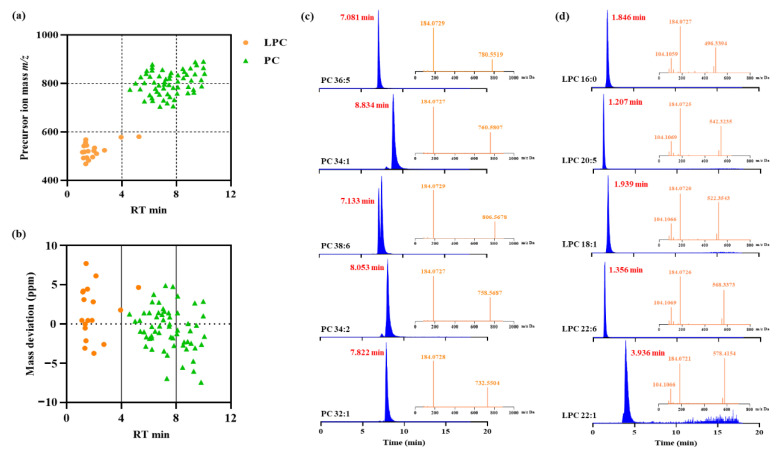
The detection of phosphatidylcholine (PC) and lyso-phosphatidylcholine (LPC) species in krill oils using the IDA mode with reversed-phase liquid chromatography. (**a**) The distribution of PC and LPC species in liquid chromatography. (**b**) The mass deviation of PC and LPC species. (**c**) Five most abundant PC species in krill oil. (**d**) Five most abundant LPC species in krill oil. The liquid chromatogram was filled with blue and the corresponding MS/MS spectrum was filled with orange.

To develop an accurate and sensitive approach for the identification of phospholipids, phospholipid-rich krill oil was used as the example in this work. Krill oil has been reported to be predominantly composed of choline-containing phospholipids [20]. It was therefore focused on for the identification of PC and LPC species. Using IDA mode, a total of 60 PC species and 18 LPC species were confirmed in krill oils (Table S2), with LPC species dominating the region from 1.0 to 4.0 min and PC species eluted from 4.5 to 10.0 min (Figure 1a). As LPC molecules only bind one fatty acyl group, strengthening molecular polarity, they elute markedly earlier than PC species in RPLC [21]. Furthermore, the distribution of mass deviation between practical mass and predicted mass was from −10 to 10 ppm (Figure 1b), indicating that identified PC and LPC species were receivable. As a result, high-resolution MS is an excellent tool for the identification of phospholipids in various samples in the absence of phospholipid standards.

As shown in Figure 1c,d, the representative extraction ion chromatography (EIC) and matching mass spectrum of PC and LPC species were exhibited, including PC 36:5, PC 34:1, PC 38:6, PC 34:2 and PC 32:1, as well as LPC 16:0, LPC 20:5, LPC 18:1, LPC 22:6 and LPC 22:1. As expected, these phospholipids have also been reported in previous studies because of their high content in krill oil [21,22]. In fact, analysis of the fragmentation spectra obtained typically uncovered the identity of the compounds with huge probability, which offered an essential insight into the discovery of novel species. For PC and LPC species, they possessed higher ionization efficiencies in positive mode as [M + H]^+^ quasi-molecular ions and produced the most dominant product ion at *m/z* 184.07, which was formed by the phosphocholine head [23]. Following this regularity, other PC and LPC species with low abundance were supposed to be found when they were acquired in SWATH mode because more comprehensive MS/MS information would be generated, even for precursor ions with feeble intensity.

### 3.2. Establishment of a Mathematical Model for PC and LPC

**Figure 2:**
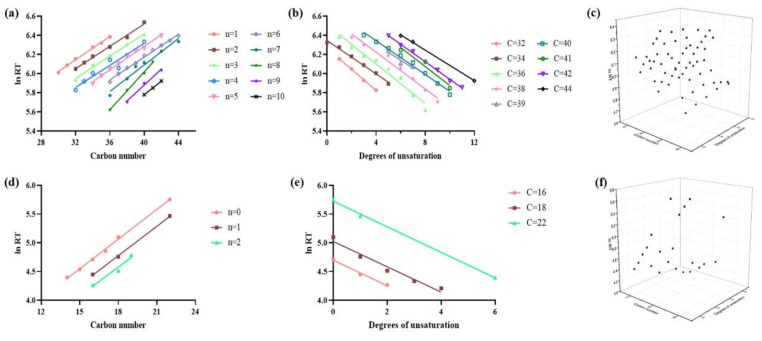
Construction of mathematical models using identified phosphatidylcholine (PC) and lyso-phosphatidylcholine (LPC) species. (**a**–**c**) Regression curves of the *ln* RT versus the carbon numbers of the fatty acyl chain, unsaturation degrees of the fatty acyl chain and the plane surface model for PC species. (**d**–**f**) Similar models for LPC species.

Since the SWATH mode weakened the strict corresponding relations between precursor ions and fragmentations, the additional RT became a suitable dimension to screen the PC or LPC candidates. In order to predict the RT of undetected PC or LPC species, a series of mathematical models were established based on the IDA-confirmed PC and LPC species (Figure 2).

The models concerning the carbon numbers of the fatty acyl chain with the same unsaturation degree showed a linear relation with the *ln* RT on the reversed phase C18 column (Figure 2a,d), and most regression coefficient values (*R*^2^) were greater than 0.95, whether in PC or LPC species (Table 1). These results demonstrate that PC and LPC species with specific double-bond numbers (n = 1–10 for PC species and n = 0–2 for LPC species) could be filtered by their RT. Similar models according to the unsaturation degrees of the fatty acyl chain with the same carbon numbers and *ln* RT were also constructed (Figure 2b,e). They also displayed linear correlations, with *R*^2^ greater than 0.95 for both PC and LPC species. In terms of these results, PC species with a carbon number of 32, 34, 36, 38, 39, 40, 41, 42 and 44, as well as LPC species with a carbon number of 16, 18 and 22, could be determined by their RT on the C18 column. Besides PC and LPC species, linear correlations between carbon number vs. retention behavior and unsaturation degree vs. retention behavior were also proven in fatty acids, fatty amines, cholesteryl esters and glycerolipids [24,25,26].

The in-depth analysis in Figure 2c,f shows three-dimensional (3D) models that were established to expand the profiling of unknown PC and LPC species. Specifically, the 3D scatter plots were plotted by integrating three variables: the carbon number, unsaturation degree and *ln* RT from the confirmed PC or LPC species. These variables were opportunely fitted with the plane regression equation, with Y = 0.062X_1_ − 0.090X_2_ + 4.216 (R^2^ = 0.9453) and Y = 0.168X_1_ − 0.225X_2_ + 2.007 (R^2^ = 0.9896) applied to PC and LPC species, respectively. In these equations, *Y* represented the retention behavior *ln* RT, *X*_1_ represented the carbon numbers, and *X_2_* represented the unsaturation degrees of the fatty acyl chain. To sum up, these models provided subsidiary conditions related to RT to recognize PC and LPC species, rather than merely depend on matching against reference MS/MS spectra.

### 3.3. Model-Assisted Identification for SWATH Results

**Figure 3:**
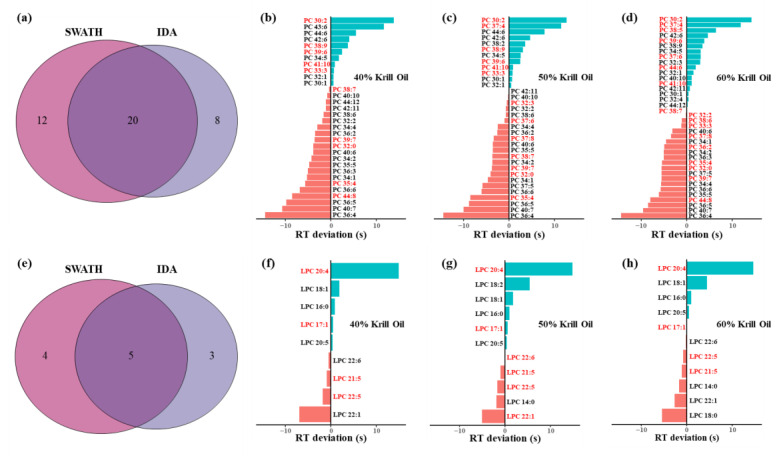
Model-assisted identification of phosphatidylcholine (PC) and lyso-phosphatidylcholine (LPC) species in krill oils. (**a**) Total identified PC species using hybrid IDA and SWATH acquisition. (**b**–**d**) The retention time (RT) deviation of PC species in different krill oils. (**e**) Total identified LPC species using hybrid IDA and SWATH acquisition. (**f**–**h**) The RT deviation of LPC species in different krill oils. The phospholipid species in black were found in both IDA and SWATH acquisition, while the species in red were only found in SWATH acquisition.

All krill oil samples with different phospholipid content were analyzed using both IDA and SWATH modes before digestion. There were 40 and 12 species of PC and LPC in the different krill oils, which contained 12 PC species and 4 LPC species only detected in the SWATH results (Figure 3a,e). These results indicated that hybrid IDA and SWATH acquisition improved detection performance by approximately 50% compared to conventional IDA acquisition, meaning more phospholipid species could be found by the newly developed method. Likewise, there have been many methods promoting detectability via improvements related to SWATH acquisition [27,28].

As for the specific phospholipid species in each krill oil group, the RT deviations between calculated values and measured values are also displayed in Figure 3. Under the condition that RT deviation was less than 15 s, there were 32 PC species and 9 LPC species in krill oil with 40% phospholipid content, 34 PC species and 11 LPC species in krill oil with 50% phospholipid content and 38 PC species and 11 LPC species in krill oil with 60%phospholipid content. Additionally, the distributions of carbon numbers and double-bond numbers of the fatty acyl chain were consistent with previous studies [17,18]. The carbon numbers were concentrated from 30 to 44 for PC species and from 16 to 22 for LPC species, while the double-bond numbers of PC and LPC species were around 4 to 12 and 0 to 6, respectively. Although our identification rested on the species level rather than molecular species level or deeper full structure level [29], more PC and LPC species were found in our results, reflecting the advantage of the developed method to some degree. Among the different krill oils, not only was the amount of identified PC and LPC species increased, but their abundances also became higher with the increase in phospholipid content (Figure S1). Especially for PC species with high unsaturation degrees (double-bond number ≥ 5), their relative abundances were distinctly higher in krill oil containing 60% phospholipids, suggesting a higher nutritive value of that krill oil [30].

### 3.4. Analysis of PC and LPC Species in the Final Digestive Products

**Figure 4:**
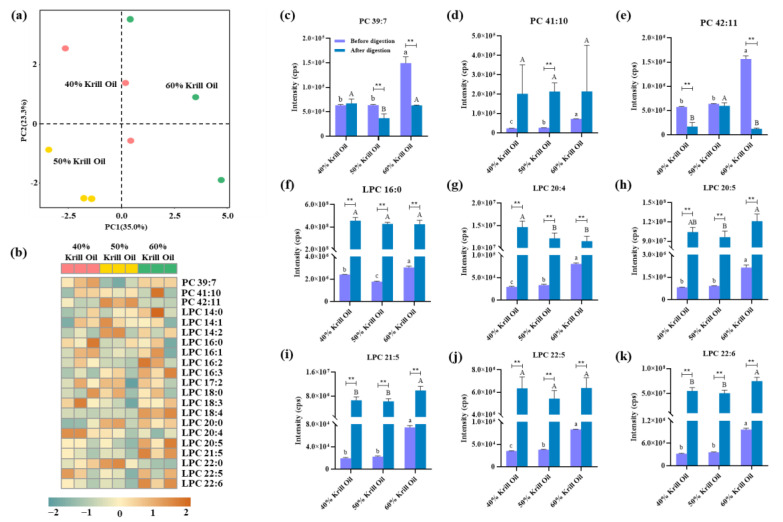
The differences between phosphatidylcholine (PC) and lyso-phosphatidylcholine (LPC) species in the final digestive products of different krill oils. (**a**) PCA score plot of identified PC and LPC species. The red points represent krill oil samples with 40% phospholipid content, the yellow points represent samples with 50% phospholipid content and the green points represent samples with 60% phospholipid content. (**b**) Heat map of identified PC and LPC species. (**c**–**k**) The alterations to PC and LPC species before and after digestion. Different lowercase letters indicate significant differences before digestion and different capital letters indicate significant differences after digestion (Duncan, *p* < 0.05). Moreover, ** indicates significant differences before and after digestion (Duncan, *p* < 0.01).

In fact, the detection of initial krill oils is just one aspect. A deeper understanding of the digestive products of krill oils is equally important. The final digestive products of different krill oils were also analyzed using the hybrid IDA and SWATH acquisition. As shown in Figure 4a, an unsupervised principal component analysis (PCA) model was built to highlight the differences between PC and LPC profiles in the final digestive products. The score plot with the first two principal components exhibits an obvious separation among different digestive products, demonstrating that initial phospholipid proportions significantly affected the composition of phospholipids in the final digestive products. Conceivably, these differences would further cause the diversity of emulsions formed by the final digestive products [31].

Focusing on the specific phospholipids, a total of 21 PC and LPC species were recognized using the developed method and relative abundances are presented in Figure 4b. In line with the results for initial krill oils, the abundances of most LPC species were increased with the rise in phospholipid content. On account of digestion, more than 90% of PC molecules were hydrolyzed, causing only three PC species (PC 39:7, PC 41:10 and PC 42:11) to be detected in the final digestive products. In contrast, more than half of the LPC species were newly emerged in the final digestive products. It could be seen that, besides free fatty acids, lyso-phospholipid was also one of the important final digestive products of phospholipid.

For detailed comparison of phospholipids before and after digestion, their MS intensities were used and are demonstrated in Figure 4c–k. In krill oil containing a 60% phospholipid proportion, the contents of PC 39:7 and PC 42:11 were significantly decreased after digestion, exactly matching the expectations. However, the change in PC 41:10 was opposite, showing a growing trend after digestion, which might be caused by the casual synthesis reaction [32]. As for the content of LPC species, they were significantly increased after digestion, no matter how much the phospholipid proportion was in initial krill oils. Notably, a lot of polyunsaturated LPC species, such as LPC 20:4, LPC 20:5 and LPC 22:6, were generated in the final digestive products, meaning they would produce different nutritional effects from the common arachidonic acid (ARA), eicosapentaenoic acid (EPA) and docosahexaenoic acid (DHA). As previously reported, polyunsaturated fatty acids formed with phospholipids were better absorbed by the intestine and readily incorporated into cell membranes compared to those formed with glycerolipids [33]. Simultaneously, the specific transporter at the blood–brain barrier was considered to transfer the lyso-phospholipid-formed DHA specifically, improving memory in adult mice [34,35]. Overall, the final digestive products of krill oil contained lots of functional LPC species, and their content usually increased with the initial phospholipid proportion.

### 3.5. Alterations in Digestive Behavior

**Figure 5:**
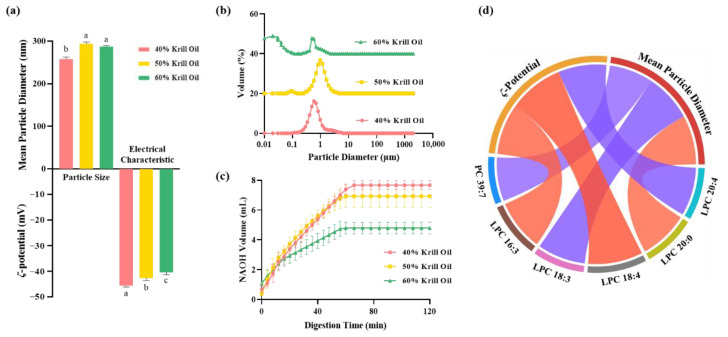
The physicochemical properties of final digestive products and correlations with phosphatidylcholine (PC) and lyso-phosphatidylcholine (LPC) species. (**a**) The mean particle diameter and *ζ*-potential of different final digestive products. (**b**) The particle size distribution of different final digestive products. (**c**) The volume of NaOH required to maintain a constant pH in emulsions containing different krill oils as measured using a pH-stat in vitro digestion model. (**d**) The Spearman correlations of particle characterization and phospholipid species, where blue lines represent negative correlation and red lines represent positive correlation. The colored cambers represent different phospholipid species or particle characterization. Different lowercase letters indicate significant differences (Duncan, *p* < 0.05).

On the one hand, the composition of PC and LPC species in the final digestive products of krill oil were analyzed; on the other hand, the physicochemical properties of final digestive products were also characterized. The mean particle diameter of all digestive product emulsions was appreciably decreased compared to the mouth phase and stomach phase (Figure 5a and Figure S2a). This is perhaps owing to the function of pancreatic lipase and bile salts, with numerous triacylglycerols and phospholipids in krill oil being digested, thus reducing the aggregation of droplets [36,37]. Comparing the mouth phase and stomach phase, the negative charge of final digestive products was also greatly increased, which could be ascribed to the generation of various anionic species, such as free fatty acids, salts, peptides, undigested lipids and proteins. All these anionic species made contributions to the electrical signal used to determine the *ζ*-potential [31,38]. In addition, the final digestive products of krill oil containing 60% phospholipid content had a bimodal particle size distribution (Figure 5b), indicating that a more complex system was generated. Meanwhile, the least consumed NaOH solution (Figure 5c) also proved this view, with residual phospholipids as the emulsifiers possibly integrated into mixed micelles, forming smaller particles [36,39].

At other stages of the simulated digestion model, the particle properties of different krill oils were also characterized to exhibit their digestive behavior more completely (Figure S2). At the initial stage, the emulsions of different krill oils were stable, with less than 200 nm of mean particle diameter and more relatively small droplets in the size distribution. In the mouth phase and stomach phase, the mean particle diameter of all emulsions was significantly increased, and the particle size distributions became bimodal trends. The *ζ*-potential exhibited a notable decrease in the magnitude of negative charge on the droplets. The whole emulsion system became unstable, which was mainly attributed to the effects of enzymes, proteins and some salt ions [40,41].

In order to reveal the potential relationships between phospholipids and particle characterizations, their correlations were evaluated using the Pearson method. The significant correlations (*p* < 0.05) are shown in Figure 5d. Whether in positive or negative, the absolute value of correlation coefficient was greater than 0.6, demonstrating that some PC and LPC species probably affect the mean particle diameter and *ζ*-potential of the final digestive products. In this regard, the LPC 16:3 and LPC 18:4 species were positively correlated with the *ζ*-potential, while the LPC 20:4 species was negative. The PC 39:7 and LPC 18:3 species were negatively correlated with the mean particle diameter, but the LPC 20:0 species was positive. These differences in correlations are likely ascribed to the particular electrical properties or aggregation behavior of PC and LPC species.

## 4. Outlook

It is necessary to acknowledge that this work is an example study focusing on the PC and LPC species in krill oils and their digestive products, neglecting other classes of phospholipids such as PE, PS, PG, PI, etc. Due to the limitation of commercially available standards, the models have not been verified by extra PC and LPC species. Nevertheless, this work proposes a pragmatic guideline for the identification of phospholipids in complex food samples. For different classes of phospholipids, they have different characteristic fragmentation for the generation of a theoretical database. In detail, PE produces the characteristic product ion [M + H-141]^+^ via elimination of the phosphoethanolamine moiety, PS produces the characteristic product ion [M + H-185]^+^ by the elimination of the phosphoserine moiety, PG produces the characteristic product ion [M + H-172]^+^ from a part of phosphoglycerol, and PI produces the characteristic product ion in the negative mode at *m/z* 241 also arising from the phosphoinositide head [42,43]. Using the computationally generated database and IDA results-based RT models, more phospholipid species were discovered by the hybrid IDA and SWATH acquisition, further improving the insights on phospholipids.

## 5. Conclusions

In summary, we have developed a new method for the analysis of phospholipids in digestion by using hybrid IDA and SWATH acquisition. This new method combines credible database identification, a model-assisted filter, LC-MS analysis and in vitro digestion. In this pilot study, we successfully identified 52 PC and LPC species in the krill oil samples, with approximately half of the PC and LPC species undetected in conventional IDA results, thus showing the huge potential of the developed method in the discovery of unknown phospholipids. Additionally, this method was applied to analyze the final digestive products of different krill oils, and 3 PC and 18 LPC species were found. LPC species not only imply the biological function of krill oil, but also partly support the alterations in digestive behaviors. This method expands the view of phospholipid digestion to the molecular level, facilitating understanding of lipid digestion and absorption.

## Figures and Tables

**Table 1:** The fitting results of different models.

**Unsaturation** **Degrees**	**Regression Equation**	**Linearity** **R^2^**	**Carbon** **Numbers**	**Regression** **Equation**	**Linearity** **R^2^**
n = 1	Y = 0.061X + 4.191	0.9960	C = 32	Y = −0.110X + 6.265	0.9990
n = 2	Y = 0.058X + 4.183	0.9938	C = 34	Y = −0.087X + 6.345	0.9910
n = 3	Y = 0.058X + 4.088	0.9915	C = 36	Y = −0.103X + 6.512	0.9654
n = 4	Y = 0.058X + 4.009	0.9159	C = 38	Y = −0.096X + 6.605	0.9792
n = 5	Y = 0.061X + 3.832	0.9893	C = 39	Y = −0.082X + 6.631	0.9051
n = 6	Y = 0.060X + 3.783	0.9942	C = 40	Y = −0.088X + 6.695	0.9875
n = 7	Y = 0.069X + 3.321	0.9694	C = 41	Y = −0.099X + 6.865	0.9508
n = 8	Y = 0.099X + 2.059	0.9976	C = 42	Y = −0.092X + 6.855	0.9963
n = 9	Y = 0.083X + 2.560	0.9919	C = 44	Y = −0.080X + 6.882	0.9989
n = 10	Y = 0.071X + 2.939	0.9999			
**Unsaturation** **Degrees**	**Regression** **Equation**	**Linearity** **R^2^**	**Carbon** **Numbers**	**Regression** **Equation**	**Linearity** **R^2^**
n = 0	Y = 0.172X + 1.965	0.9975	C = 16	Y = −0.223X + 4.698	0.9883
n = 1	Y = 0.171X + 1.700	0.9990	C = 18	Y = −0.221X + 5.023	0.9656
n = 2	Y = 0.166X + 1.583	0.9547	C = 22	Y = −0.223X + 5.724	0.9979
**Regression Equation**	**Linearity R^2^**	**Regression Equation**		**Linearity R^2^**	
Y = 0.062X_1_ − 0.090X_2_ + 4.216	0.9453	Y = 0.168X_1_ − 0.225X_2_ + 2.007		0.9896	

Y: *ln* RT, *X*_1_: carbon numbers of the fatty acyl chain, *X*_2_: unsaturation degrees of the fatty acyl chain.

## Data Availability

The data presented in this study are available on request from the corresponding author.

## Supplementary Materials

The following supporting information can be downloaded at: https://www.mdpi.com/article/10.3390/foods12102020/s1, Figure S1: Abundance of phosphatidylcholine (PC) and lyso-phosphatidylcholine (LPC) species in different krill oils with 40%, 50% and 60% of phospholipid contents; Figure S2: The digestive behaviors of krill oils with 40%, 50% and 60% of phospholipid contents after exposure to different stages of simulated digestion model; Table S1: Lower and upper m/z limits of the SWATH precursor selection windows used; Table S2: Identified phosphatidylcholine (PC) and lyso-phosphatidylcholine (LPC) species using IDA mode.
