# Supplementary material for: Analysis of Phospholipids in Digestion Using Hybrid IDA and SWATH Acquisition: An Example for Krill Oil
Source: Foods. 2023 May 16;12(10):2020. doi: 10.3390/foods12102020 (PMC10217430; doi:10.3390/foods12102020)
Supplement: Supplementary file 1 [file foods-12-02020-s001.zip › foods-2355828-supplementary.pdf]

Supplement

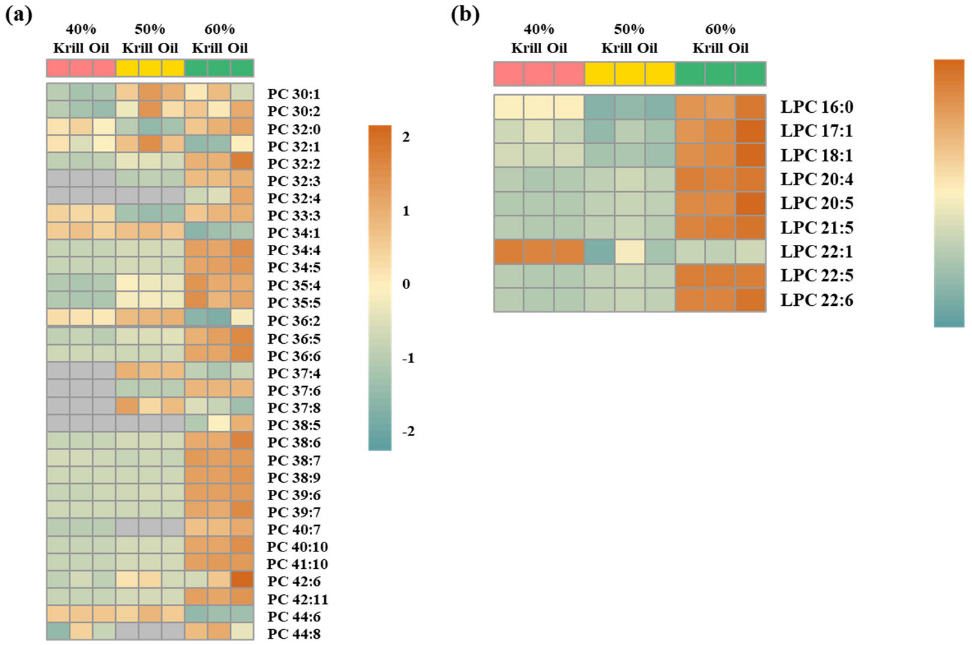

Figure S1. Abundance of phosphatidylcholine (PC) and lyso-phosphatidylcholine (LPC) species in different krill oils with 40%, 50% and 60% of phospholipid contents. (a) Heat map of PC species. (b) Heat map of LPC species.

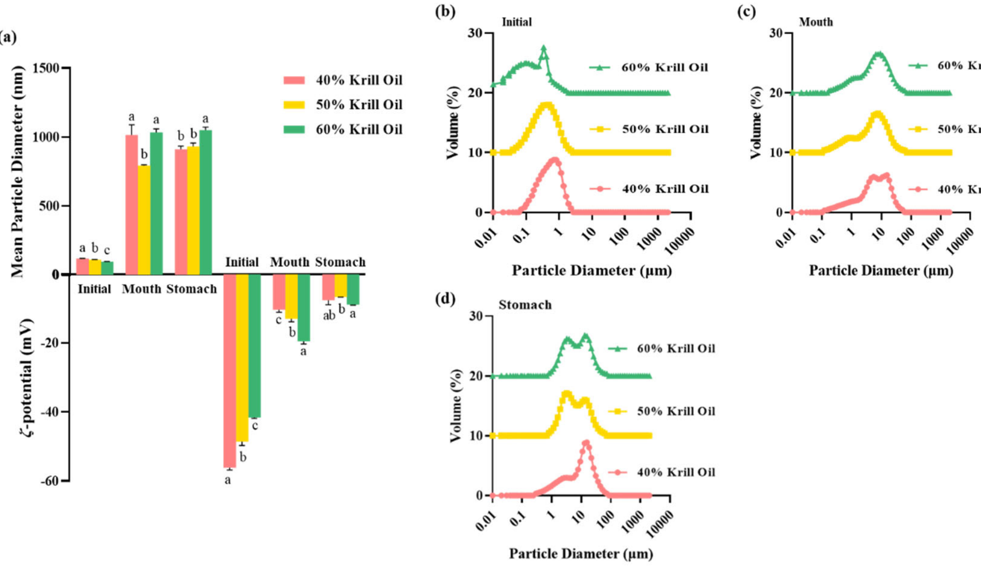

Figure S2. The digestive behaviors of krill oils with 40%, 50% and 60% of phospholipid contents after exposure to different stages of simulated digestion model. (a) Mean particle diameter and electrical characteristics (ζ-potential) of different krill oils. (b)-(d) Particle size distribution of different krill oils. Different lowercase letters indicated significant differences (Duncan, $p < 0.05$).

Table S1. Lower and upper *m/z* limits of the SWATH precursor selection windows used

**Experiment**	**Lower *m/z* limit**	**Upper *m/z* limit**
1	100	1000
2	100	160
3	159	220
4	219	280
5	279	340
6	339	400
7	399	460
8	459	520
9	519	580
10	579	640
11	639	700
12	699	760
13	759	820
14	819	880
15	879	940
16	939	1000

Table S2. Identified phosphatidylcholine (PC) and lyso-phosphatidylcholine (LPC) species using IDA mode

Compound	Molecular formula	Retention time RT min	Practical mass *m/z* [a]	Predicted mass *m/z* [b]	Mass deviation ppm [c]
PC 30:0	$C_{38}H_{76}O_8NP$	7.768	706.5415	706.5381	4.77
PC 30:1	$C_{38}H_{74}O_8NP$	6.791	704.5223	704.5225	-0.26
PC 31:1	$C_{39}H_{76}O_8NP$	7.322	718.5331	718.5381	-6.94
PC 32:1	$C_{40}H_{78}O_8NP$	7.843	732.5518	732.5538	-2.76
PC 32:2	$C_{40}H_{76}O8NP$	7.052	730.5396	730.5381	1.94
PC 32:3	$C_{40}H_{74}O_8NP$	6.254	728.5245	728.5225	2.83
PC 32:4	$C_{40}H_{72}O_8NP$	5.653	726.5061	726.5068	-1.00
PC 33:2	$C_{41}H_{78}O_8NP$	7.526	744.5538	744.5538	-0.01
PC 33:4	$C_{41}H_{74}O_8NP$	6.203	740.5214	740.5225	-1.51
PC 34:0	$C_{42}H_{84}O_8NP$	9.283	762.5961	762.6007	-6.03
PC 34:1	$C_{42}H_{82}O_8NP$	8.849	760.5834	760.5851	-2.25
PC 34:2	$C_{42}H_{80}O_8NP$	8.050	758.5685	758.5694	-1.25
PC 34:3	$C_{42}H_{78}O_8NP$	7.352	756.5508	756.5538	-3.97
PC 34:4	$C_{42}H_{76}O_8NP$	6.754	754.5373	754.5381	-1.11
PC 34:5	$C_{42}H_{74}O_8NP$	6.041	752.5236	752.5225	1.44
PC 35:1	$C_{43}H_{84}O_8NP$	9.252	774.5971	774.6007	-4.75
PC 35:5	$C_{43}H_{76}O_8NP$	6.572	766.5388	766.5381	0.90
PC 36:1	$C_{44}H_{86}O_8NP$	9.857	788.6143	788.6164	-2.61
PC 36:2	$C_{44}H_{84}O_8NP$	8.871	786.6008	786.6007	0.05
PC 36:3	$C_{44}H_{82}O_8NP$	8.100	784.5845	784.5851	-0.78
PC 36:4	$C_{44}H_{80}O_8NP$	7.765	782.5681	782.5694	-1.76
PC 36:5	$C_{44}H_{78}O_8NP$	7.066	780.5542 [a]	780.5538	0.54
PC 36:6	$C_{44}H_{76}O_8NP$	6.460	778.5374	778.5381	-0.92
PC 36:7	$C_{44}H_{74}O_8NP$	5.348	776.5228	776.5225	0.45
PC 36:8	$C_{44}H_{72}O_8NP$	4.598	774.5078	774.5068	1.27
PC 37:4	$C_{45}H_{82}O_8NP$	7.112	796.5823	796.5851	-3.44
PC 37:5	$C_{45}H_{80}O_8NP$	7.452	794.5706	794.5694	1.42
PC 37:6	$C_{45}H_{78}O_8NP$	6.711	792.5549	792.5538	1.45
PC 38:2	$C_{46}H_{88}O_8NP$	9.827	814.6260	814.6320	-7.43
PC 38:3	$C_{46}H_{86}O_8NP$	9.097	812.6144	812.6164	-2.46

PC 38:5	$C_{46}H_{82}O_8NP$	8.103	808.5862	808.5851	1.43
PC 38:6	$C_{46}H_{80}O_8NP$	7.133	806.5707	806.5694	1.62
PC 38:7	$C_{46}H_{78}O_8NP$	6.368	804.5568	804.5538	3.78
PC 38:8	$C_{46}H_{76}O_8NP$	5.658	802.5393	802.5381	1.40
PC 38:9	$C_{46}H_{74}O_8NP$	5.006	800.5228	800.5225	0.36
PC 39:5	$C_{47}H_{84}O_8NP$	8.512	822.5985	822.6007	-2.70
PC 39:6	$C_{47}H_{82}O_8NP$	7.494	820.5848	820.5851	-0.29
PC 39:7	$C_{47}H_{80}O_8NP$	7.223	818.5734	818.5694	4.89
PC 40:3	$C_{48}H_{90}O_8NP$	10.064	840.6464	840.6477	-1.57
PC 40:4	$C_{48}H_{88}O_8NP$	9.369	838.6343	838.6320	2.68
PC 40:5	$C_{48}H_{86}O_8NP$	8.757	836.6137	836.6164	-3.19
PC 40:6	$C_{48}H_{84}O_8NP$	8.113	834.6037	834.6007	3.56
PC 40:7	$C_{48}H_{82}O_8NP$	7.532	832.5858	832.5851	0.89
PC 40:8	$C_{48}H_{80}O_8NP$	6.744	830.5717	830.5694	2.76
PC 40:9	$C_{48}H_{78}O_8NP$	6.067	828.5523	828.5538	-1.85
PC 40:10	$C_{48}H_{76}O_8NP$	5.389	826.5374	826.5381	-0.94
PC 41:6	$C_{49}H_{86}O_8NP$	8.588	848.6144	848.6164	-2.29
PC 41:8	$C_{49}H_{82}O_8NP$	7.617	844.5837	844.5851	-1.66
PC 41:10	$C_{49}H_{78}O_8NP$	5.784	840.5514	840.5538	-2.84
PC 42:5	$C_{50}H_{90}O_8NP$	9.989	864.6486	864.6477	1.02
PC 42:6	$C_{50}H_{88}O_8NP$	9.034	862.6313	862.6320	-0.86
PC 42:7	$C_{50}H_{86}O_8NP$	8.478	860.6116	860.6164	-5.52
PC 42:9	$C_{50}H_{82}O_8NP$	6.976	856.5861	856.5851	1.21
PC 42:10	$C_{50}H_{80}O_8NP$	6.211	854.5679	854.5694	-1.76
PC 42:11	$C_{50}H_{78}O_8NP$	5.820	852.5524	852.5538	-1.65
PC 43:6	$C_{51}H_{90}O_8NP$	9.489	876.6475	876.6477	-0.18
PC 43:11	$C_{51}H_{80}O_8NP$	6.226	866.5692	866.5694	-0.32
PC 44:6	$C_{52}H_{92}O_8NP$	9.989	890.6659	890.6633	2.87
PC 44:7	$C_{52}H_{90}O_8NP$	9.382	888.6450	888.6477	-3.06
PC 44:12	$C_{52}H_{80}O_8NP$	6.234	878.5666	878.5694	-3.23
LPC 14:0	$C_{22}H_{46}O_7NP$	1.352	468.3087	468.3085	-0.53
LPC 15:0	$C_{23}H_{48}O_7NP$	1.558	482.3239	482.3241	0.44
LPC 16:0	$C_{24}H_{50}O_7NP$	1.854	496.3395	496.3398	0.46
LPC 16:1	$C_{24}H_{48}O_7NP$	1.423	494.3203	494.3241	7.71
LPC 16:2	$C_{24}H_{46}O_7NP$	1.187	492.3065	492.3085	4.08

LPC 17:0	$C_{25}H_{52}O_7NP$	2.142	510.3523	510.3554	6.13
LPC 18:0	$C_{26}H_{54}O_7NP$	2.727	524.3724	524.3711	-2.61
LPC 18:1	$C_{26}H_{52}O_7NP$	1.940	522.3539	522.3554	2.83
LPC 18:2	$C_{26}H_{50}O_7NP$	1.523	520.3375	520.3398	4.44
LPC 18:3	$C_{26}H_{48}O_7NP$	1.269	518.3225	518.3241	3.11
LPC 18:4	$C_{26}H_{46}O_7NP$	1.119	516.3082	516.3085	0.46
LPC 19:2	$C_{27}H_{52}O_7NP$	1.998	534.3574	534.3554	-3.74
LPC 20:4	$C_{28}H_{50}O_7NP$	1.410	544.3409	544.3398	-2.15
LPC 20:5	$C_{28}H_{48}O_7NP$	1.214	542.3218	542.3241	4.20
LPC 21:5	$C_{29}H_{50}O_7NP$	1.335	556.3415	556.3398	-3.09
LPC 22:0	$C_{30}H_{62}O_7NP$	5.254	580.4310	580.4337	4.65
LPC 22:1	$C_{30}H_{60}O_7NP$	3.942	578.4170	578.4180	1.78
LPC 22:6	$C_{30}H_{50}O_7NP$	1.350	568.3398	568.3398	-0.02

[a] The practical mass was detected in positive ionization mode for $[M+H]^+$ adduct.

[b] The predicted mass was obtained from OS-Q software according to the molecular formula.

[c] The mass deviation was calculated by equation: (Practical mass - Predicted mass)/ Predicted mass.
